# Ultrasonographic assessment of abnormal fetal growth related to uteroplacental-fetal biometrics and Doppler (U-AID) indices: Protocol for multicenter retrospective cohort study trial

**DOI:** 10.1371/journal.pone.0298060

**Published:** 2024-02-15

**Authors:** Eun-Saem Choi, Hwasun Lee, Se Jin Lee, Young Mi Jung, Ho Yeon Kim, Seung Mi Lee, Kyung A. Lee, Hyun-Joo Seol, Hyun Sun Ko, Sung Hun Na, Dong Wook Kwak, Han-Sung Hwang, Sooran Choi, Soon-Cheol Hong, Hye-Sung Won, Suk Young Kim, Hai-Joong Kim, Ki Hoon Ahn

**Affiliations:** 1 Department of Obstetrics and Gynecology, Korea University College of Medicine, Korea University Anam Hospital, Seoul, Korea; 2 Department of Biostatistics, Korea University College of Medicine, Seoul, Korea; 3 Department of Obstetrics and Gynecology, School of Medicine Kangwon National University, Chuncheon-si, Gangwon-Do, Korea; 4 Department of Obstetrics and Gynecology, Seoul National University College of Medicine, Seoul, Korea; 5 Department of Obstetrics and Gynecology, Korea University Ansan Hospital, Korea University College of Medicine, Ansan, Korea; 6 Department of Obstetrics and Gynecology, Ewha Womans University School of Medicine, Seoul, Korea; 7 Department of Obstetrics and Gynecology, Kyung Hee University School of Medicine, Seoul, Korea; 8 Department of Obstetrics and Gynecology, College of Medicine, The Catholic University of Korea, Seoul, Korea; 9 Department of Obstetrics and Gynecology, Ajou University School of Medicine, Suwon, Korea; 10 Department of Obstetrics and Gynecology, Konkuk University Medical Center, Konkuk University School of Medicine, Seoul, Korea; 11 Department of Obstetrics and Gynecology, College of Medicine, Inha University, Incheon, Korea; 12 Department of Obstetrics and Gynecology, Asan Medical Center, University of Ulsan College of Medicine, Seoul, Korea; 13 Department of Obstetrics and Gynecology, Gachon University Gil Hospital, Incheon, South Korea; Aga Khan University - Kenya, KENYA

## Abstract

Fetal growth restriction (FGR) is one of the leading causes of perinatal morbidity and mortality. Many studies have reported an association between FGR and fetal Doppler indices focusing on umbilical artery (UA), middle cerebral artery (MCA), and ductus venosus (DV). The uteroplacental-fetal circulation which affects the fetal growth consists of not only UA, MCA, and DV, but also umbilical vein (UV), placenta and uterus itself. Nevertheless, there is a paucity of large-scale cohort studies that have assessed the association between UV, uterine wall, and placental thickness with perinatal outcomes in FGR, in conjunction with all components of the uteroplacental-fetal circulation. Therefore, this multicenter study will evaluate the association among UV absolute flow, placental thickness, and uterine wall thickness and adverse perinatal outcome in FGR fetuses. This multicenter retrospective cohort study will include singleton pregnant women who undergo at least one routine fetal ultrasound scan during routine antepartum care. Pregnant women with fetuses having structural or chromosomal abnormalities will be excluded. The U-AID indices (UtA, UA, MCA, and UV flow, placental and uterine wall thickness, and estimated fetal body weight) will be measured during each trimester of pregnancy. The study population will be divided into two groups: (1) FGR group (pregnant women with FGR fetuses) and (2) control group (those with normal growth fetus). We will assess the association between U-AID indices and adverse perinatal outcomes in the FGR group and the difference in U-AID indices between the two groups.

## Introduction

Fetal growth restriction (FGR), which is found in approximately 3–8% of pregnancies, is a leading cause of perinatal morbidity and mortality [1, 2]. The definition of FGR differs according to clinical guidelines and author groups [3–5]. The International Society of Ultrasound in Obstetrics and Gynecology (ISUOG) defines FGR as a pathologic condition in which the fetus cannot reach its own determined genetic growth. According to the (ISUOG) practice guidelines, the finding of an abnormal uterine artery (UtA) or umbilical artery (UA) on Doppler examination should be accompanied by an abdominal circumference (AC) or estimated fetal weight (EFW) of less than the 10^th^ percentile for gestational age to diagnose early FGR unless the AC or EFW is less than the 3^rd^ percentile [5]. In contrast, the American College of Obstetricians and Gynecologists (ACOG) and the Society for Maternal-Fetal Medicine (SMFM) define FGR as a fetus whose AC or EFW is less than the 10^th^ percentile [2, 3].

Even though the definition of FGR may defer between guidelines, consistently worse perinatal outcomes of FGR fetuses with abnormal UA Doppler results have been reported compared to those with normal UA Doppler results [6–9]. Therefore, fetal vascular Doppler examinations, including UA Doppler, are important in distinguishing a pathologically growth-restricted fetus from a small but healthy fetus. Several studies predicted the perinatal outcomes of fetuses with growth restriction and these studies mainly focused on the Doppler examination of specific vessels, such as the UA, MCA, or ductus venosus (DV) [6–12]. However, besides the UA, MCA, or DV, uteroplacental-fetal circulation also consists of other components affecting fetal growth. For example, umbilical vein (UV) flow and placental thickness, which are components of uteroplacental-fetal circulation, have already been shown to be associated with fetal growth. Ferrazzi et al. reported that UV flow per unit head circumference (HC) was decreased in FGR fetuses [13]. Another study reported that venous flow was also reduced in FGR fetuses [14]. In a previous study assessing the correlation between placental thickness and small for gestational age (SGA) neonates, the placental thickness-to-EFW ratio was higher in SGA neonates compared to non-SGA neonates [15]. In addition, a thick placenta was reported to be associated with adverse perinatal outcomes [16]. In this research, we aim to focus on the uterine wall, in conjunction with the umbilical vein. The uterine wall is a distinctive aspect of this study, as it has been neglected in previous research on FGR. The causes of a thick uterine wall observed in ultrasonography are typically categorized into three main factors: sustained uterine wall contractions, adenomyosis, and uterine fibroids [17, 18]. If a thick uterine wall is observed in a pregnant woman without a history of uterine fibroids or adenomyosis, it may be attributed to a sustained uterine contraction. The uterine contractions are known to lead to a decrease in uteroplacental blood flow [18–20]. Taking this into consideration, uterine wall thickness may be associated with the uteroplacental blood flow. The hypothesis that uterine wall thickness may impact fetal growth is one of the key starting points for our research.

Therefore, we aim to evaluate the association of UV absolute flow, placental thickness, and uterine wall thickness with adverse perinatal outcomes in the FGR fetuses, ultimately seeking to validate their involvement in the pathophysiology of FGR. In addition, we will comprehensively assess various uteroplacental-fetal biometrics and Doppler indices according to fetal growth in each trimester of pregnancy to establish a prediction model for adverse perinatal outcomes in FGR fetuses using these ultrasonographic measurements.

## Methods

### Study design and population

The U-AID study is a multicenter retrospective cohort study which is led by an obstetric ultrasonography research society of Korean Society of Ultrasound in Obstetrics and Gynecology (KSUOG). This is conducted in 13 academic hospitals in the Republic of Korea. This study includes singleton pregnant women who undergo fetal ultrasound during routine antepartum surveillance. The eligible participants are pregnant women over 19 years old who can understand information about the current study and make decisions on whether to voluntarily participate in the study. There is no limitation on gestational age but pregnant women with fetuses found to have structural anomalies, abnormal insertion of umbilical cord, chromosomal abnormalities, or any kind of congenital infection will be excluded. The abnormal insertion of umbilical cord includes velamentous insertion and marginal insertion of umbilical cord. Of note, pregnant women with missing value of UV flow, placental thickness, and uterine wall thickness will be excluded.

### Inclusion criteria

- Singleton pregnant women
- Age older than 19 years
- Participants who have no difficulty understanding information on the current study so they can voluntarily decide to participate and provide fully informed consent
- Receiving fetal ultrasound for routine antepartum surveillance

### Exclusion criteria

- Pregnant women with missing value of UV flow, placental thickness, and uterine wall thickness
- Pregnant women with fetuses with possible structural anomalies or abnormal insertion of umbilical cord found on ultrasound
- Pregnant women with fetuses with chromosomal abnormalities
- Pregnant women who are proven to have an intrauterine infection
- Pregnant women who have limited data on clinical characteristics related to pregnancy and perinatal outcome

In this study, we define FGR as a status where both of the following 2 conditions are met; (1) an EFW or AC below the 10th percentile for gestational age during the second or third trimester of pregnancy and (2) birthweight below the 10th percentile for gestational age [3]. The INTERGROWTH-21^st^ growth chart is used for biometry and birthweights [21]. The FGR group includes both early-onset FGR and late-onset FGR. The control group includes participants with a fetus whose EFW and AC are in the range of 10^th^ to 90^th^ percentile for gestational age. The control group will be selected through 1:1 matching based on age, parity, and presence or absence of preeclampsia. The clinical information and U-AID indices of pregnant women who delivered between March 2016 and March 2023 will be extracted from the medical records.

### Sample size

We calculated the minimum number of FGR participants using PASS (NC, USA). Based on a published study, we set an α-error of 0.05 and a β-error of 80%, and the minimum number of FGR participants required was 472. Considering a follow-up loss rate of 10%, we need to enroll at least 519 pregnant women with FGR fetuses. Therefore, the minimum number of total participants that we anticipate including is 1,050 (525 FGR participants and 525 control participants). The researchers will first review the medical records and include eligible participants retrospectively.

### Outcomes

The primary outcome is the association of UV absolute flow, placental thickness, and uterine wall thickness with adverse perinatal outcomes in the FGR group to differentiate genuinely growth-restricted fetuses from those who are constitutionally small. The secondary outcome is to evaluate the differences in U-AID indices (UtA, UA, MCA, and UV flow, placental and uterine wall thickness, and estimated fetal body weight) between the FGR and control group will be evaluated.

### Ultrasonographic uteroplacental-fetal biometrics and Doppler (U-AID) indices

We will collect the ultrasonographic uteroplacental-fetal biometric measurements and U-AID indices listed in Table 1 in each trimester of pregnancy. During the first trimester of pregnancy, we will collect ultrasonographic measurements from 11^0/7^ to 13^6/7^ weeks of gestation. The measurements will be assessed from 18^0/7^ to 23^6/7^ weeks of gestation during the second trimester of pregnancy and from 30^0/7^ to 36^6/7^ weeks of gestation during the third trimester of pregnancy. The ultrasound model used and the clinical experience years of clinicians who measure U-AID indices will also be documented. To assess fetal growth, crown to rump length, biparietal diameter, head circumference, abdominal circumference, femur length, and estimated body weight will be evaluated. UA Doppler which was measured in a free cord loop and MCA Doppler which was measured in the proximal third of the MCA will be selected. The systolic/diastolic ratio (S/D ratio), pulsatile index (PI), and resistive index (RI) in the UtA, UA, MCA, and UV will be calculated as reported previously [22, 23]. The Doppler indices will be calculated as follows.

- S/D ratio = peak systolic velocity [cm/sec] (S)–end-diastolic velocity [cm/sec] (D)
- PI = S–D / mean of S and D (M)
- RI = S–D / S
- Notch index = early diastolic flow velocity [cm/sec]/ peak diastolic flow velocity [cm/sec]
- UV absolute flow [ml/min] = UV cross-sectional area [cm^2^] * UV mean velocity [cm/sec] * 60

**Table 1:** Ultrasonographic uteroplacental-fetal biometric measurements and Doppler indices of the U-AID cohort.

**Uteroplacental-fetal biometry**	**Timing of measurement**	**Unit**
Crown to rump length	First trimester	Mm
Biparietal diameter	Second trimester, Third trimester	cm
Head circumference	Second trimester, Third trimester	cm
Abdominal circumference	Second trimester, Third trimester	cm
Femur length	Second trimester, Third trimester	cm
Estimated fetal weight	Second trimester, Third trimester	g
Placental thickness	Second trimester, Third trimester	mm
Uterine wall thickness	First trimester, Second trimester, Third trimester	mm
**Uteroplacental-fetal Doppler**		**Indices**
Uterine artery	First trimester, Second trimester, Third trimester	S/D ratio, PI, RI, early diastolic notch index
Umbilical artery	Second trimester, Third trimester	S/D ratio, PI, RI
Middle cerebral artery	Second trimester, Third trimester	S/D ratio, PI, RI
Ductus venosus	Second trimester, Third trimester	S/D ratio, PI, RI
Umbilical vein	Second trimester, Third trimester	Pulsation, cross-sectional diameter, mean velocity, absolute flow

Abbreviation: PI, pulsatile index; RI, resistive index; S/D ratio, systolic/diastolic ratio

**Fig 1:**
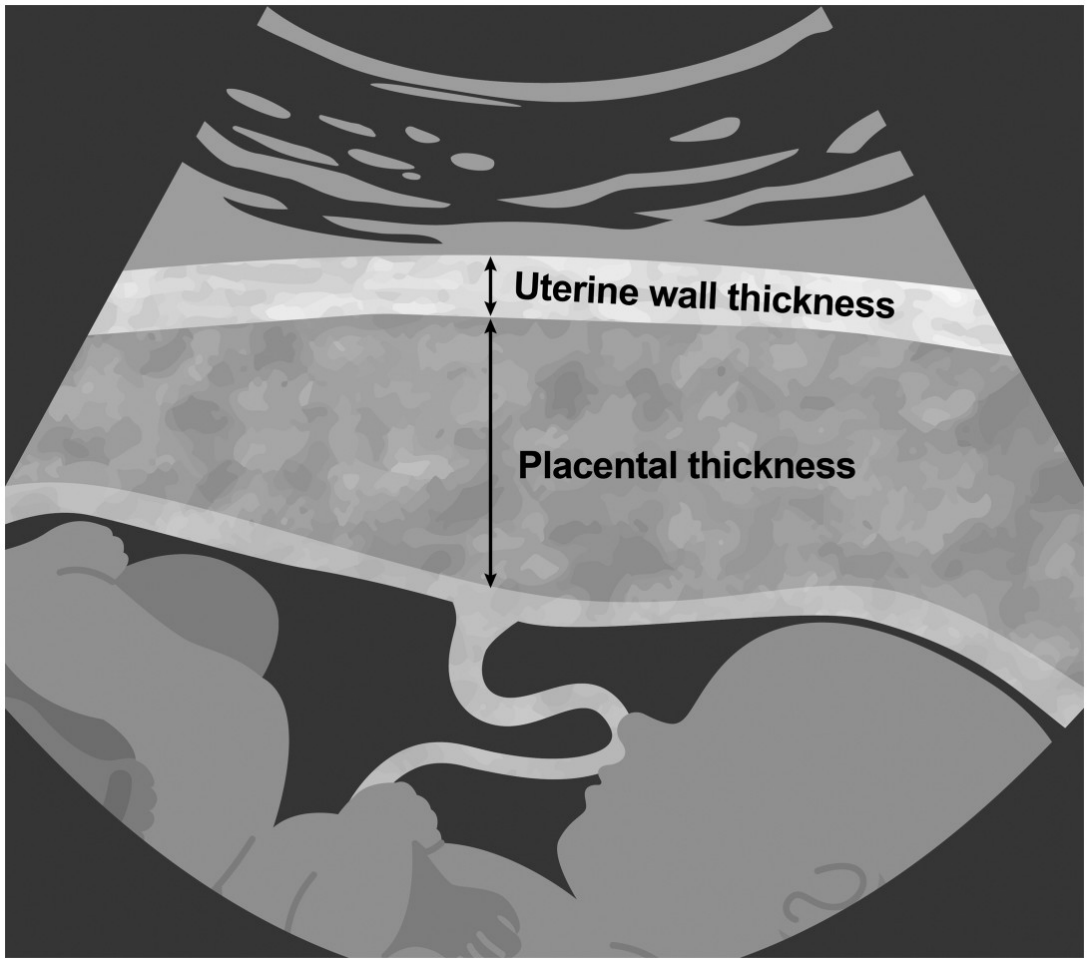
The measurement of placental and uterine wall thickness.

The placental thickness and the uterine wall thickness which were measured through the sagittal section at the location of cord insertion will be collected. (Fig 1). The uterine wall was considered as a homogenous layer above the placenta.

### Adverse perinatal outcomes

Pregnancy and perinatal outcomes will be acquired from medical charts. The adverse perinatal outcomes of the current study are listed in Table 2. The composite morbidity of neonates is defined as one of the following: sepsis, respiratory distress syndrome, necrotizing enterocolitis, or intraventricular hemorrhage before discharge.

**Table 2:** Adverse perinatal outcomes in the U-AID cohort.

**Pregnancy outcomes**
Preeclampsia
Emergency cesarean section due to non-reassuring fetal heartbeat
Intrauterine fetal demise
**Neonatal outcomes**
Birthweight
Admission to neonatal intensive care unit (NICU),
Sepsis before discharge
Respiratory distress syndrome before discharge
Necrotizing enterocolitis before discharge
Intraventricular hemorrhage before discharge
Periventricular leukomalacia
Neonatal death before discharge

### Data management

The study will be managed according to General Data Protection Regulations and Good Clinical Practice (GCP). Data will be collected securely on case report forms (CRFs) and each participant will be assigned a unique number for pseudonymization. All study data will be stored securely on password-protected PCs and only accessible to the researchers of the current study. Paper CRFs, paper consent forms, and any paper records of study data will be kept in locked drawers or cabinets in a secure location.

### Statistical methods

The distribution of U-AID indices according to AC, BPD, EFW and gestational age will be assessed. The study population will be divided into two groups, the FGR group which includes participants with FGR fetuses, and the control group includes participants with normal growth fetus. First of all, we will compare the differences in U-AID indices according to adverse perinatal outcomes with in FGR group. Secondly, the difference of U-AID indices between the two groups will be compared. Continuous variables will be presented as the mean ± standard deviation or median [interquartile range] and compared by the Mann-Whitney U test or independent t-test according to the distribution of each variable. Categorical variables will be presented as the number of observations (percent) and compared by the chi-squared test or Fisher’s exact test. To determine the contribution of all the collected variables and Doppler findings to adverse perinatal outcomes of the FGR fetuses, multivariable logistic regression analysis with backward stepwise elimination will be used.

All statistical analyses will be performed using IBM SPSS statistics version 23.0 (SPSS Inc., Chicago, IL, USA) and R version 4.2.1 (http://www.r-project.org).

## Ethics and dissemination

The current study received ethics approval from the Institutional Review Board (IRB) of Korea University Anam Hospital (2015AN0083).

## Discussion

The novelty of this study lies in its comprehensive examination of all the parts within the pathway of blood flow from mother to fetus. The rationale behind this study design stems from the objective to investigate the pathophysiology of fetal growth within the clinically observable range of ultrasonography, encompassing the processes that involve UtA, uterine wall, placenta, UV and fetal growth, The aim is to elucidate the pathophysiology of fetal growth. Through this study, we aim to find the association between new ultrasonographic indices, including UV absolute flow, and placental and uterine wall thickness and perinatal outcome of growth restricted fetuses. As it is significant to distinguish growth restricted fetuses with adverse perinatal outcomes from the constitutionally small fetuses, we posit that this study may contribute to the identification of new risk factors or the development of a prediction model for genuinely growth restricted fetuses in future study. By evaluating and comparing the various ultrasonographic uteroplacental-fetal biometric measurements and Doppler indices according to fetal growth, we can determine how much each ultrasonographic index influences fetal growth. Through this finding, we can provide evidence for future studies on the pathophysiology of FGR fetuses. Lastly, we can also assess the distribution of umbilical vein flow values according to estimated fetal weight in each trimester of pregnancy.

There are some possible limitations of this study. Firstly, because this is a multicenter cohort study, the quality of ultrasound examinations may vary from center to center. However, because in all participating hospitals, the ultrasonography is performed only by obstetricians, we posit that a certain degree of quality control measures could be instituted. Secondly, the lack of long-term outcomes on FGR fetuses is another limitation of the study. Thirdly, the lack of information regarding contraction at the time of measurement is another limitation of the current study. The presence or absence of uterine contractions can affect the measurement of the uterine wall thickness. However, because of the inherent limitation of retrospective study, we consider that accurately determining the presence or absence of contractions at the time of measurement is unfeasible. Lastly, because the uteroplacental-feta Doppler waveforms are not usually measured in a routine perinatal check of fetuses with normal EFW, a certain degree of missing values is anticipated within the context of this study. To address this aspect, a large-scale prospective cohort study will be necessary in the future.

Current studies have mostly focused on the UA or MCA, and even though large-scale multicenter Doppler studies have been conducted, there is no general consensus on the optimal timing for the delivery of compromised FGR fetuses. This study may lay the foundation for future studies to suggest the optimal timing for the best perinatal outcomes of FGR fetuses.

## References

[1] Romo A, Carceller R, Tobajas J. Intrauterine growth retardation (IUGR): epidemiology and etiology. Pediatr Endocrinol Rev. 2009;6 Suppl 3:332–6. . 19404231

[2] Society for Maternal-Fetal Medicine (SMFM), Martins JG, Biggio JR, Abuhamad A. Society for Maternal-Fetal Medicine Consult Series #52: Diagnosis and management of fetal growth restriction: (Replaces Clinical Guideline Number 3, April 2012). Am J Obstet Gynecol. 2020;223(4):B2–17. Epub 20200512. doi: 10.1016/j.ajog.2020.05.010 . 32407785

[3] American College of Obstetricians and Gynecologists (ACOG) Committee. Fetal Growth Restriction: ACOG Practice Bulletin, Number 227. Obstet Gynecol. 2021;137(2):e16–28. doi: 10.1097/AOG.0000000000004251 . 33481528

[4] Lausman A, Kingdom J. Intrauterine growth restriction: screening, diagnosis, and management. J Obstet Gynaecol Can. 2013;35(8):741–8. doi: 10.1016/S1701-2163(15)30865-3 . 24007710

[5] Lees CC, Stampalija T, Baschat A, da Silva Costa F, Ferrazzi E, Figueras F, et al. ISUOG Practice Guidelines: diagnosis and management of small-for-gestational-age fetus and fetal growth restriction. Ultrasound Obstet Gynecol. 2020;56(2):298–312. doi: 10.1002/uog.22134 . 32738107

[6] Unterscheider J, Daly S, Geary MP, Kennelly MM, McAuliffe FM, O’Donoghue K, et al. Optimizing the definition of intrauterine growth restriction: the multicenter prospective PORTO Study. Am J Obstet Gynecol. 2013;208(4):290.e1–6. doi: 10.1016/j.ajog.2013.02.007 . 23531326

[7] Ghosh GS, Gudmundsson S. Uterine and umbilical artery Doppler are comparable in predicting perinatal outcome of growth-restricted fetuses. BJOG. 2009;116(3):424–30. doi: 10.1111/j.1471-0528.2008.02057.x . 19187375

[8] Spinillo A, Montanari L, Roccio M, Zanchi S, Tzialla C, Stronati M. Prognostic significance of the interaction between abnormal umbilical and middle cerebral artery Doppler velocimetry in pregnancies complicated by fetal growth restriction. Acta Obstet Gynecol Scand. 2009;88(2):159–66. doi: 10.1080/00016340802632358 . 19169929

[9] Tolu LB, Ararso R, Abdulkadir A, Feyissa GT, Worku Y. Perinatal outcome of growth restricted fetuses with abnormal umbilical artery Doppler waveforms compared to growth restricted fetuses with normal umbilical artery Doppler waveforms at a tertiary referral hospital in urban Ethiopia. PLoS One. 2020;15(6):e0234810. Epub 20200618. doi: 10.1371/journal.pone.0234810 . 32555633 PMC7302535

[10] Lees C, Marlow N, Arabin B, Bilardo CM, Brezinka C, Derks JB, et al. Perinatal morbidity and mortality in early-onset fetal growth restriction: cohort outcomes of the trial of randomized umbilical and fetal flow in Europe (TRUFFLE). Ultrasound Obstet Gynecol. 2013;42(4):400–8. doi: 10.1002/uog.13190 . 24078432

[11] Walker DM, Marlow N, Upstone L, Gross H, Hornbuckle J, Vail A, et al. The Growth Restriction Intervention Trial: long-term outcomes in a randomized trial of timing of delivery in fetal growth restriction. Am J Obstet Gynecol. 2011;204(1):34.e1–9. Epub 20101105. doi: 10.1016/j.ajog.2010.09.019 . 21056403

[12] Turan OM, Turan S, Berg C, Gembruch U, Nicolaides KH, Harman CR, et al. Duration of persistent abnormal ductus venosus flow and its impact on perinatal outcome in fetal growth restriction. Ultrasound Obstet Gynecol. 2011;38(3):295–302. doi: 10.1002/uog.9011 . 21465604

[13] Ferrazzi E, Rigano S, Bozzo M, Bellotti M, Giovannini N, Galan H, et al. Umbilical vein blood flow in growth-restricted fetuses. Ultrasound Obstet Gynecol. 2000;16(5):432–8. doi: 10.1046/j.1469-0705.2000.00208.x . 11169327

[14] Boito S, Struijk PC, Ursem NT, Stijnen T, Wladimiroff JW. Umbilical venous volume flow in the normally developing and growth-restricted human fetus. Ultrasound Obstet Gynecol. 2002;19(4):344–9. doi: 10.1046/j.1469-0705.2002.00671.x . 11952962

[15] Ahn KH, Lee JH, Cho GJ, Hong SC, Oh MJ, Kim HJ. Placental thickness-to-estimated foetal weight ratios and small-for-gestational-age infants at delivery. J Obstet Gynaecol. 2017;37(7):883–7. Epub 20170620. doi: 10.1080/01443615.2017.1312306 28631507

[16] Miwa I, Sase M, Torii M, Sanai H, Nakamura Y, Ueda K. A thick placenta: a predictor of adverse pregnancy outcomes. Springerplus. 2014;3:353. Epub 20140711. doi: 10.1186/2193-1801-3-353 . 25077064 PMC4112033

[17] Kido A, Togashi K. Uterine anatomy and function on cine magnetic resonance imaging. Reprod Med Biol. 2016;15(4):191–9. Epub 20160213. doi: 10.1007/s12522-016-0235-y . 29259437 PMC5715863

[18] Togashi K, Kawakami S, Kimura I, Asato R, Takakura K, Mori T, et al. Sustained uterine contractions: a cause of hypointense myometrial bulging. Radiology. 1993;187(3):707–10. doi: 10.1148/radiology.187.3.8497617 . 8497617

[19] Cramer SF, Heller DS. A Review and Reconsideration of Nonneoplastic Myometrial Pathology. Int J Surg Pathol. 2018;26(2):104–19. Epub 20171218. doi: 10.1177/1066896917748194 . 29254394

[20] Inoue A, Horinouchi T, Yoshizato T, Kojiro-Sanada S, Kozuma Y, Ushijima K. Peculiar blood flow profiles among placental chorionic villous vessels of an abnormally thick placenta in a case of systemic lupus erythematosus characterized using microvascular imaging. J Obstet Gynaecol Res. 2020. Epub 20201012. doi: 10.1111/jog.14502 . 33047457

[21] Papageorghiou AT, Ohuma EO, Altman DG, Todros T, Cheikh Ismail L, Lambert A, et al. International standards for fetal growth based on serial ultrasound measurements: the Fetal Growth Longitudinal Study of the INTERGROWTH-21st Project. Lancet. 2014;384(9946):869–79. doi: 10.1016/S0140-6736(14)61490-2 . 25209488

[22] Callen PW, Norton ME, Scoutt LM, Feldstein VA. Callen’s ultrasonography in obstetrics and gynecology. 6th ed. Philadelphia PA: Elsevier; 2016.

[23] Park YW, Cho JS, Choi HM, Kim TY, Lee SH, Yu JK, et al. Clinical significance of early diastolic notch depth: uterine artery Doppler velocimetry in the third trimester. Am J Obstet Gynecol. 2000;182(5):1204–9. doi: 10.1067/mob.2000.104840 . 10819859

